# Stress and the City: Mental Health in Urbanized vs. Rural Areas in Salzburg, Austria

**DOI:** 10.3390/ijerph21111459

**Published:** 2024-10-31

**Authors:** Vanessa Natalie Frey, Patrick Benjamin Langthaler, Martin Josef Huf, Günter Gruber, Thomas Prinz, Ludmilla Kedenko, Bernhard Iglseder, Bernhard Paulweber, Eugen Trinka

**Affiliations:** 1 Department of Neurology, Neurointensive Care and Neurorehabilitation, European Reference Network EpiCARE, Christian Doppler University Hospital, Centre for Cognitive Neuroscience, Paracelsus Medical University Salzburg, 5020 Salzburg, Austria; 2 Department of Artificial Intelligence and Human Interfaces, Paris Lodron University of Salzburg, 5020 Salzburg, Austria; 3 Team Biostatistics and Big Medical Data, IDA Lab Salzburg, Paris Lodron University of Salzburg, 5020 Salzburg, Austria; 4 Department of Public Health, Health Services Research and Health Technology Assessment, UMIT—University for Health Sciences, Medical Informatics and Technology, 6060 Tyrol, Austria; 5 Research Studio iSPACE, Research Studios Austria Forschungsgesellschaft mbH, 5020 Salzburg, Austria; 6 Team Space & Mobility, IDA Lab Salzburg Paris Lodron University Salzburg, 5020 Salzburg, Austria; 7 Department of Internal Medicine, St. Johanns University Hospital, Private Medical University of Salzburg, 5020 Salzburg, Austria; 8 Department of Geriatric Medicine, Christian Doppler University Hospital, Paracelsus Medical University, 5020 Salzburg, Austria

**Keywords:** depression, Paracelsus 10,000, green space, noise pollution, population density, migration

## Abstract

Background: Living in the city is associated with a higher risk of suffering from stress, anxiety, and depression. Due to an increase of migration to the city, the association between mental health and city life is highly relevant to society. Methods: We analyzed data of 9573 participants (Ø 55.3 years, SD = 7.4) of the Paracelsus 10,000 study (Salzburg, Austria) who were classified into having or not having depressive symptoms. Population density, green space, and noise around the home address of the participants were collected and tested for correlations with mental health defined by depressive symptoms. We additionally tested whether migration status influenced the effect of urbanization on mental health. Results: There is a positive correlation between degree of urbanization and the probability of suffering from depressive symptoms (*p* = 0.011), yet this effect is independent of the migration background (*p *= 0.581). Participants in areas with high residential density were significantly more likely to suffer from poor mental health (*p *= 0.006 unadjusted). No significant association could be found between mental health and noise (*p* = 0.126 unadjusted) or green space neither regarding distance to closest green space (*p* = 0.549 unadjusted), nor size of green space (*p* = 0.549 unadjusted). Conclusions: In the Paracelsus 10,000 cohort, living in the city is associated with lower mental health, especially in participants with a high population density in the direct neighborhood. This might be due to social stress yet does not reflect minority stress in migrants. However, the influence of noise pollution and green space on mental health is limited in this cohort.

## 1. Introduction

The relationship between mental health and urbanization is a crucial and prevailing issue due to an increasing population living in urban areas [1]. In 1950, 30% of the world population lived in cities, while in 2014, 54% did so, and predictions estimate that in the year 2050 two-thirds of the world population will live in urban areas [1]. According to the American Psychological Association Task Force on Urban Psychology, people living in cities cope with more pollution, violence, crimes, and crowding than people living in rural areas [2,3]. These factors are all associated with higher risk of stress, anxiety, and depression [2,3]. Several studies uncovered a significantly lower quality of life in patients with depression compared to the healthy population or compared to individuals with chronic medical disorders [4,5,6]. A review published in 1974 revealed higher rates of psychiatric disorders in urban areas [7], especially for mood disorders and anxiety disorders [8]. Further studies showed that living in a city is one of the most predictable factors for developing major psychiatric disorders like schizophrenia [9,10]. A Danish study on patients with schizophrenia uncovered a dose-dependent effect: individuals who lived in a highly urbanized area during the first 15 years of their life show a 2.75 times higher prevalence to develop schizophrenia [11].

The underlying drivers for this remain unknown [12], yet there are suggestions that air pollution, traffic noise, small housing, social stress, and the restricted availability of green space might be factors influencing mental health in people living in urban regions and with this quality of life [3,4,12,13,14,15,16]. Several studies demonstrated an inverted association between traffic noise and mental health [14,17]. In a German study, researchers analyzed data of participants who developed depressive symptoms between the first study phase and the follow up 5 years later. They observed a correlation between the development of depressive symptoms and the exposure to traffic noise. This effect was strongest in participants with low socioeconomic status and those who experience sleep disturbances [13]. A review of 31 studies dealing with the influence of traffic noise on mental health found a positive association between aircraft noise and the risk of developing depression [18]. However, the authors found no significant increase in risk of suffering from depression due to road and railway traffic noise. Klompmaker and colleagues suggest that the influence of traffic noise, air pollution, and green space on mental health when observed in isolation may be overestimated, while the effect of these three factors in combination have a higher impact than expected [17]. Hence, the association between mental health and traffic noise is not clear yet.

Green space in urban settings has been shown to have a positive influence on mental health [12,15,16,19,20]. This was also observed via biomarkers for stress and attention [21], like brain waves measured via portable electroencephalography devices during a walk in green zones [22] or while watching photographs of landscapes [23]. The results showed a reduction of stress and mental fatigue during the walk in the green space or while watching photographs of meadows and forests. A further biomarker for stress, diurnal variation of salivary cortisol, was shown to be reduced in individuals living in a neighborhood with surrounding green space [24,25]. Another study indicates a dose-dependent effect of green space: The probability of suffering from mental health issues was 55% higher in the Danish population who grew up with the lowest level of green space compared to those who grew up with the highest level of green space [12]. Even though there are numerous studies on the influence of green space on mental health, it is difficult to draw a general conclusion due to heterogeneous methods of data acquisition and analysis throughout these studies [19,26].

The social environment is a further issue contributing to the influence of urban living on mental health [8,27]. Social support and social interaction might be reduced by insecurity, violence, and disorganized neighborhoods in urban areas [3]. While the interaction within the local community (the bonding social capital) might be higher in rural areas, the interaction between the local community and people outside the local community (the bridging social capital) is marginally higher in urban regions [28]. This might lead to a more open-minded attitude towards minorities and outsiders in the city, while in rural areas more social discrimination can be observed [3]. Especially minorities like immigrants might be negatively affected when living in rural areas and suffer from social exclusion leading to “minority stress”. This in turn negatively affects mental health and well-being [3]. Not only social isolation, but also social stress can lead to mental health problems, which are believed to be higher in the city [29,30]. This might be caused by a crowded environment, the feeling of lack of privacy, exposure to strangers, and an unclear dominance order [31,32,33,34].

Studies on Austrian populations regarding urbanization and mental health have not been published so far. However, quality of life was analyzed in one district of Salzburg, an Austrian city with a population of 158,040 individuals (January 2024) [35,36]. The study uncovered positive correlations between quality of life and low population density, as well as with living in small houses with gardens. While in a further study conducted in six districts in Salzburg (1/3 of the whole city), the housing satisfaction and “feeling at home” was shown to be important for quality of life [37]. Contrary to global trends, the inhabitants of Salzburg tend to emigrate from the city to the surrounding areas. According to current data, especially the age group from 20 to 29 leave the city, possibly to start a family [38]. In the Paracelsus 10,000 study, more than 10,000 randomly chosen inhabitants of Salzburg City and the surrounding areas were investigated [39]. We collected data on health status, lifestyle, and genetics. For the data analysis in this work, we focused on mental health and its association with the degree of urbanization and the constitution of the direct neighborhood within this population. We hypothesize that the study participants living in the city show a higher prevalence of poor mental health than participants living in rural areas. Additionally, we suggest that this depends on the following factors: residential density, amount of green space, and noise pollution in the direct neighborhood. We also intend to uncover the relationship between migration background, mental health, and urbanization in the Salzburg population.

This issue is of high relevance as it shows possible points of action in improving city design and the distribution of mental health care facilities [8]. Additionally, it helps to understand the emergence of mental health issues in cities and provides background for early prevention strategies. Not many such studies have been conducted in the Austrian population, uncovering a possible blind spot in case management.

## 2. Methods

### 2.1. The Paracelsus 10,000 Cohort

We used data from the Paracelsus 10,000 study, an epidemiological study that investigated the health status of 10,000 inhabitants of the Salzburg City, Austria, and the surrounding areas. The aim of the study is to analyze the prevalence of cardiovascular, cerebrovascular, as well as metabolic and psychological diseases and correlate them with lifestyle and genetic components. Participants were aged between 40 and 77 years and were randomly selected from the local population registry [39]. The first recruitment phase of the study was conducted between the years 2013 and 2020 including a medical prevention program, intensified functional testing, and inventories on lifestyle and sociodemographic data. For detailed information on all investigations, see Frey et al. [39]. The study was approved by the local ethics committee (Land Salzburg: 415-E/1521/3-2012), and all participants signed an informed consent form. For this evaluation, we used the address of the participants, as well as their self-reported information on mental health and demographics (sex, age, migration background, and educational status). As confounders we included the health status of the participants involving the following diseases: diabetes, coronary heart diseases, chronic heart failure, arterial obstructive disease, stroke, COPD, chronic renal insufficiency, chronic hepatitis, cirrhosis of the liver, rheumatoid arthritis, duodenum/gastrointestinal tumor, Alzheimer diseases or dementia, and cancer. A score was used, consisting of three levels: (1.) none of the listed diseases, (2.) one or two of the listed diseases, or (3.) three or more of the listed diseases. Participants were considered to have migration backgrounds if they or one of their parents were not born in Austria. We made an exception for Germany or Switzerland, reason being that the language as well as the culture are very similar to the ones in Austria. Additionally, Salzburg is located directly at the border to Germany, and cross-border commuters are very common (e.g., living in Germany, working in Austria). Classification into different degrees of urbanization was conducted via the degree of urbanization (DEGURBA) criteria by Eurostat from 2018 [40]. Urbanized areas are defined as having at least 50,000 inhabitants, intermediate areas having 5000 inhabitants, and rural areas less than this.

### 2.2. Inventory on Mental Health

Participants were categorized into “no depression” or “depression” according to the results of the following inventories on depressive symptoms:

- Beck Depression inventory II (BDI-II) [41]. A score higher than 13 = “depression” or lower/equal 13 = “no depression”. Cronbach’s alpha for this test was calculated to be 0.90.
- Whooley Questions (Two-Question Test) on depressive disorder [42]. Both questions must be answered with yes. Cronbach’s alpha for this test was calculated to be 0.39.

If one of the two assessments were positive, the participant was included in the group with “depression”. Please notice that the tools applied screen for depressive symptoms and are not suitable to diagnose Major Depression. We use the phrases “no depression” and “depression” to simplify the text.

### 2.3. Green Space, Residential Density, and Noise Data

**Figure 1:**
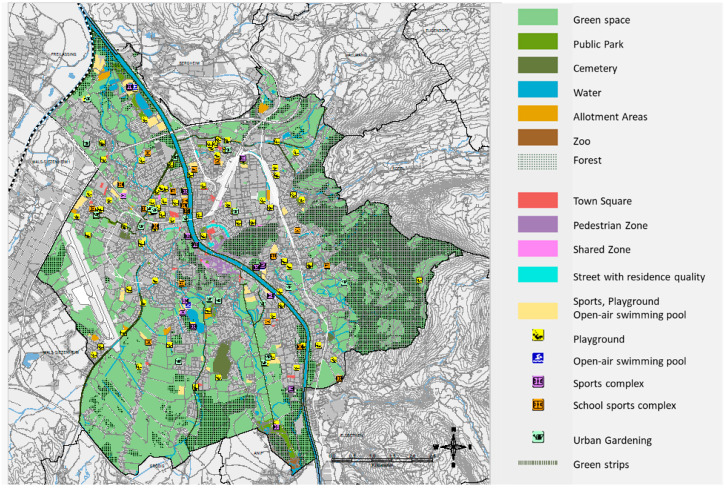
Distribution of green and free space structures in Salzburg City according to municipality Magistrat Salzburg, SAGIS, urban planning, traffic, and urban park office, BMK Land Salzburg (as at 15 June 2021) [38].

Data on green space were obtained from the state government (Land Salzburg) including rural areas, allotment areas, recreational areas, campsites, sport facilities, playgrounds, open-air swimming pools, cemeteries, waters, distance space, emission control strips, and other areas not designated as building land or traffic areas like forests (see Figure 1). To analyze the effect of green space, we calculated (1.) the distance between the address of the participant and the closest green space and (2.) the area of green space within a 300 m radius surrounding the address of the participant. Figure 1 shows green and free space in Salzburg City.

Residential density was defined to be the number of inhabitants that lived within the same 100 m × 100 m square as the participant. The aggregated registration data were provided by the municipality of the Salzburg Province.

**Figure 2:**
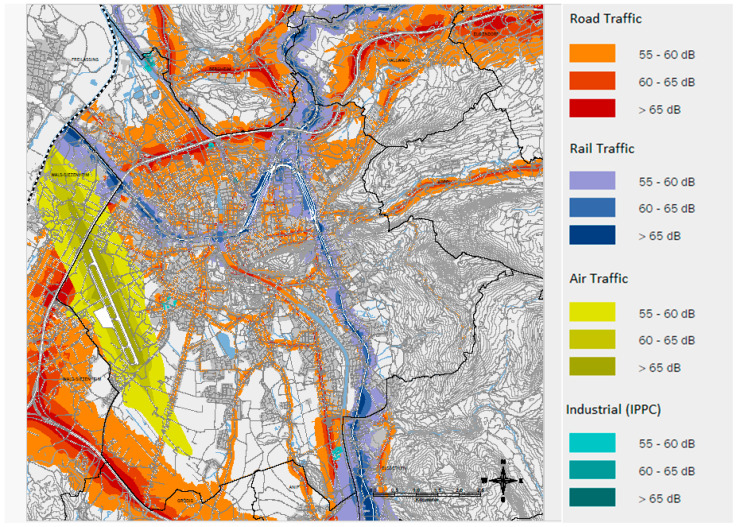
Distribution of noise levels in Salzburg City (24 h averaged) according to the municipality Magistrat Salzburg, SAGIS, urban planning, and traffic office, BMK Land Salzburg (as at 31 May 2017) [38].

Noise data were provided by the Federal Ministry Republic of Austria (Climate Action, Environment, Energy, Mobility, Innovation, and Technology) and were publicly available online [43]. The dataset is composed of (A) traffic noise caused by cars, trucks, and motorcycles, (B) noise caused by trains, (C) noise caused by airplanes, and (D) industrial noise. The data were not measured but calculated considering traffic levels, traffic composition, speed of vehicles, road surfaces, population data, and models of the terrain. This calculation model is called Common Noise assessment methods (CNOSSOS-EU) and was developed by the European Commission in 2012 [44]. Within the EU-states these methods are mandatory to calculate noise along high-level infrastructure and in urban areas. Noise load was defined as high if the sound level from any source exceeded 55 dB. Figure 2 shows the noise map of Salzburg City.

### 2.4. Statistics

Statistical analysis was conducted using the statistical software package R (version 4.2.2) [45]. All models used were logistic regression models with the binary variable of mental health (1 = “depression”, 0 = “no depression”) as the outcome. As a first preliminary step, a model was fit with the degree of urbanization of the participant’s address as predictor (Model 0a). Next, we checked for an interaction between degree of urbanization and presence of migration background (Model 0b). Since there was no significant interaction, we proceeded as follows: For each of the predictors’ Green Distance, Green Area, Population Density, and Noise, we fit a univariate model (Models 1a to 1d). Next, we fit a model including all four predictors simultaneously (Model 2). Lastly, we added all other covariables into this model (Model 3). The generalized variance inflation factor in Model 3 was below 1.7 for all variables, indicating that no substantial multicollinearity was present. For our main analysis we used multiple imputation with 20 imputed datasets, as implemented in the R package mice [46] to deal with missing values. We also used Firth correction as implemented in the package logistf [47] to obtain less biased results. In the Appendix A we present results of a sensitivity analysis in which we left out the imputation step (i.e., complete case analysis), the Firth correction or both, resulting in three alternative ways to calculate the models for each model. This sensitivity analysis was not conducted for the models 0a and 0b, where only complete case analysis was used.

Whenever multiple imputation was used, we used Rubin’s rules [48] to obtain estimates and confidence intervals for the regression parameters.

Regarding the adjustment of type 1 error, we combined Bonferroni’s method [49] with a multivariate approach based on the joint multivariate distribution of the regression estimates, implemented in the package multcomp [50]. Correction was performed simultaneously for all urbanization parameters in each of the Models 1, 2, and 3. This was performed for each of the four analyses (main analysis and the three sensitivity analyses) separately. As a cutoff for statistical significance, we chose an alpha of 0.05.

## 3. Results

### 3.1. Description of the Study Cohort

Of the 10,044 participants of the Paracelsus 10,000 study, 471 individuals had to be excluded from data analysis due to technical problems in translating the address to coordinates. We included 9573 participants with an average age of 55.26 years (SD = 7.42). Details on the study cohort and distribution of the three urbanization degrees are displayed in Table 1.

### 3.2. Degree of Urbanization and Migration Background

**Figure 3:**
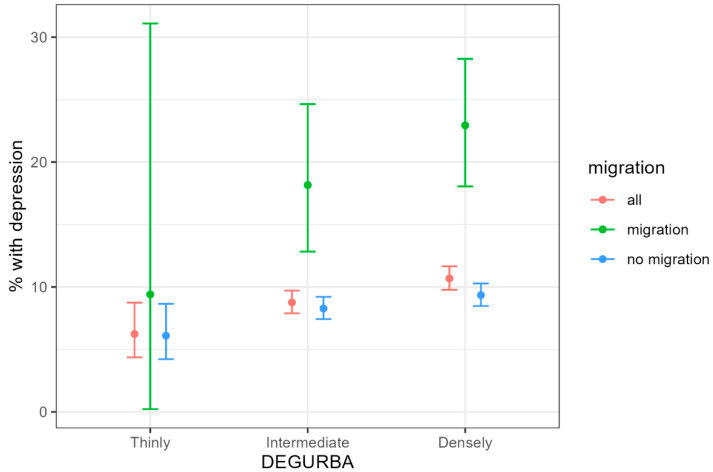
Percentage of study participants that suffer from depressive symptoms categorized in DEGURBA and with or without migration background. DEGURBA = Degree of Urbanization.

There is a positive correlation between degree of urbanization and the probability of suffering from depressive symptoms (*p* = 0.011). Study participants with migration backgrounds are more likely to suffer from depressive symptoms (*p* =< 0.001) compared to participants without migration backgrounds, yet this effect is independent of the degree of urbanization (*p* = 0.581), see Figure 3.

### 3.3. Green Space, Population Density, and Noise Pollution

The variables noise and green distance do not show significant effects in any of the models that were calculated, while population density stays significant in all models (for details see Table 2, Table 3 and Table 4). However, this effect does not survive adjustment for error 1. The variable green area only shows a significant effect in model 1, hence without taking the other variables or confounders into consideration. This effect withstands adjusting for error 1. Descriptive statistics of these variables are displayed in Table 5.

## 4. Discussion

In this work, we analyzed data from the Paracelsus 10,000 study, an epidemiological study on the health of the inhabitants of and around Salzburg City in Austria. We hypothesized a correlation between mental health (represented by the prevalence of depressive symptoms) and urbanization that emerges from the factors noise, population density, and green space availability around the participants’ residence. To exclude possible confounders, we controlled for sociodemographic as well as clinical characteristics of the study population. Positive and significant correlations were found between mental health and a higher age, being male, an income of more than 2000 Euro/month (netto per household), living with a partner, no migration background, and having no comorbidities. These correlations are well described in the literature [51,52,53,54,55,56,57,58], yet do not always agree with our outcome. Numerous studies and meta-analysis confirm that age is a risk factor for depression [59,60]. However, the population of the Paracelsus 10,000 study shows a clearly decreased risk of depressive symptoms from 60 years on. We assume this might be due to the already excluded effects of low income, educational level, living situation (alone or with partner), and comorbidities in the correlation between age and depressive symptoms. Hence, the main influencing factors of deteriorating mental health with higher age might be omitted [61]. In addition, Blazer and colleagues mentioned that even though the elderly might be of higher risk to develop depression due to biological factors, they might in return hold protective properties from a social perspective like socioemotional selectivity and relativism of values and life priorities [61].

We found differences in mental health regarding urbanization degree: Participants living in urbanized areas were more likely to suffer from depressive symptoms than participants living in rural areas. This result supports our first hypothesis and corroborates the previous studies performed in different settings that were presented in the introduction. However, our intention was to uncover which aspects of city life influence psychological health. When controlling for the other urbanization parameters our data show a significant correlation only between depressive symptoms and population density 100 m around the main residence of each participant. Hence, it might be the high number of people living in our surroundings that bothers us. This is rather surprising as humans are regarded as social beings that rely on cooperation with others to survive [62]. There is the idea of higher efficiency if people are living together, and according to this hypothesis, as a city grows, these benefits increase [63]. However, there seems to be a point at which increasing population density turns into disadvantage. In a study in the USA, a decrease in happiness could be observed with increasing size of a city or town and reaches a significant level when the population size reaches several hundred thousand [63].

What happens when the advantages of living with other individuals are displaced by the discomfort of being surrounded by too many people, for example in a crowd? According to the stimulus overload model, people suffer from an information overload when one person is too close to another [64,65]. The model suggests that qualitative and quantitative changes take place like higher information load, e.g., more facial details of more people. This information needs to be processed, and decisions have to be made rapidly in order to access and react to the situation [64,65]. Information overload can influence psychophysiological effects that might lead to behavioral and somatic pathology [66]. Lederbogen and colleagues associated city life and urban upbringing with neural social stress [27]. In their study, participants living in urban and rural areas conducted a social stress task and were investigated with magnetic resonance imaging (MRI). The results showed increased activity of the amygdala in participants living currently in the city compared to participants living in the countryside, which might reflect increased exposure to stress. Additionally, they found increased activity in the perigenual anterior cingulate cortex in participants who grew up in the city. This brain region is associated with the regulation of the amygdala, negative affect [67], and stress [68]. The results of a further MRI study suggest a link between urban upbringing and differential amygdala activation. The authors discuss that this is due to altered mechanisms of feedback learning [69].

Besides the stimulus overload in crowds, social life might be different between urban and rural areas. In his review on urbanization and emerging mental health issues, Ventriglio and colleagues found difficulties in maintaining effective social interaction in urban areas. They suggest this is due to the rhythm of city life, daily commitments, longer distances, and higher average of time spent traveling [3]. The authors also report a higher accessibility to the internet in the city resulting in more time spent online with social networks in contrast to meeting people in person [70]. Tönnis stated that cities exemplify a mechanical society without much community [71]. These factors might all contribute to an afflicting social life in the city and with this lead to reduced mental health.

However, we did not find significant effects between depressive symptoms and the size of green space or distance to the next green space. We speculate that due to the quick access to the Alps and recreation areas outside the city, the impact of green space at the place of residence is reduced. According to Statistik Austria, 45% of Austrians reported to go for a walk or hiking at least several times a month [72,73]. Additionally, three of the five most popular sports activities of Austrians are activities in the mountains. A study on Austrian mountain exercisers showed that they have a lower probability of suffering from mental health issues [74]. It is not only the physical exercise that influences mental health, but also the outdoor activity itself [75]. Hence, this effect might outperform the influence of reduced green space in the city.

Surprisingly the percentage of people living with a noise level higher than 55 dB was highest in the thinly urbanized area, and there is a trend towards higher mental health in participants living in a neighborhood with higher noise level. These effects were not significant though. We consider that houses in small villages might be rather gathered around bigger roads that connect the cities for a better infrastructure, resulting in higher noise pollution. It also has to be mentioned that the noise inside a building might be independent of the noise outside. Neighbors next door or of a flat on the same floor might be very noisy while there is hardly any traffic outside. Also, the noise data we consulted for this analysis are composed of industrial and traffic noise exclusively. However, noise caused by other sources like playgrounds, nightlife, or animals on a farm might also have an influence. Hence, analyzing the effect of noise at the place of residence is a challenging undertaking and should be considered carefully.

Our findings do not show differences between study participants with or without migration backgrounds regarding the effect of urbanization on mental health. Hence, our data do not reflect the “minority stress” that might be caused by social exclusion in rural areas. We know that individuals with migration backgrounds are underrepresented in the Paracelsus 10,000 cohort, which is especially the case in the city [39]. We assume that individuals with migration backgrounds who participated in the study are to some extent integrated in Austrian society (better language skills and cultural understanding) and might therefore not suffer from minority stress to the same extent as those who did not follow our invitation. Also, the small number of participants with migration backgrounds living in rural areas gives a very low power to detect an interaction effect involving rural areas. The sub-cohort of participants with migration backgrounds are a heterogeneous group. They migrated from diverse countries for different reasons (safety, education, partnership). We did not further categorize participants with migration backgrounds according to these parameters, nor did we take the duration of their stay into consideration. This might have an impact on the relationship between mental health, city living, and minority stress.

## 5. Limitations

Due to the observational nature of this study, the results show associations but cannot claim causality, while some further limitations must be taken into consideration:

It was argued in the literature that the lower prevalence of depressive disorders in rural areas might be due to limited access to mental health care services and surveillance compared to urban areas. Hence, in rural areas depressive disorders might be under-detected [76]. Though it has to be considered that all participants of this study followed our invitation to attend the project and were willing to visit the clinic for the investigation. Hence, access possibility and motivation were given. However, individuals with strong major depression might not have been able to attend the study, resulting in a bias in the cohort. The information about mental health was partly collected in questionnaires filled out by the study participants. Individuals that are non-native speakers might have had trouble understanding the questions or did not fill out the inventories at all. The data on mental health represent the situation of the last weeks before the visit, and hence, psychological disorders in the past are not taken into consideration, as well as the place of residence before participation in the study. The participants of this study were aged between 40 and 77, and hence the influence of city life on mental health in children, adolescents, and elderly was not tested in this study, and for this, the results are not representative for the whole population. A further limitation of the study is that the data of the 10,000 participants were collected between the years 2013 and 2020, while the report containing the noise data was generated in 2017, data on green space was created in 2021, and data on the population density in 2022. We used Open Street maps^®^ to translate addresses into coordinates, yet approximately 10% of house numbers were not recognized by the system, and the coordinate was set in the middle of the street. This might slightly influence the outcome of this analysis.

Further analysis on the relationship between urbanization and mental health is necessary to confirm the results of this study. It should be focused on a more detailed subdivision of noise compounds (traffic noise, industrial noise, noise within the housing complex). This also applies for green space. Jimenez and colleagues suggest that different types of green spaces might have different relevance to mental health, i.e., parks have a higher influence than playgrounds, cemeteries, or golf courses [77]. Also, the background and history of migration of participants should be considered for future analysis.

## 6. Conclusions

As expected, we found an association between the degree of urbanization and the prevalence of depressive symptoms in the population of Salzburg and the surrounding area. Individuals living in urban areas are more likely to suffer from depressive symptoms compared to individuals living in rural areas. Interestingly, this effect is not stronger in migrants, questioning the estimated minority stress and representativeness of the migrant population in the study cohort. The results show a negative influence of higher population density in the neighborhood on mental health. This is supported by data of former studies uncovering higher stress levels and altered brain activity in individuals living or growing up in cities. Surprisingly, the influence of noise level and green space on mental health is limited. We suggest this might be due to the special location of Salzburg, embedded in the Alps, offering a compensation of city life. This possibility is widely used by the Salzburg population and reduces stress levels. The results of this data analysis proceed to further highlight the social life in the city and its underestimated influence on mental health. Prevention strategies could be based on the one hand on avoiding large, narrow housing estates to impede a densely crowded living environment. On the other hand, the quality of social life in these estates could be improved by organizing collective leisure activities like physical exercise or street festivals to counteract social isolation and create a bond between people in the direct neighborhood. Additionally, an increased number of mental health care facilities should be provided, especially in areas with high population density. The living situation and social life of patients should be focused on when screening for mental health issues. Growing up in large housing estates and suffering from social isolation might be a biomarker for developing depressive symptoms and could be accordingly prevented. Further research on this issue is of high relevance as depression is among the largest single causes of disability worldwide [78], and a deeper understanding of its development is crucial.

## Figures and Tables

**Table 1:** Shows the socioeconomic and clinical characteristics of the study cohort, as well as the degree of urbanization. Number and percentage of participants included in each category are presented. Green area, distance to next green space, and number of inhabitants are displayed as mean with standard deviation.

Category	Variable	Level	N Participants (%)
Sociodemographic	Age	40–50	2587 (27.0)
		50–60	4195 (43.8)
		60–70	2791 (29.2)
	Sex	Female	4950 (51.7)
		Male	4623 (48.3)
	Education	ISCED 0–2	704.3 (7.4)
		ISCED 3–5	5941.8 (62.1)
		ISCED 6–8	2774.5 (29.0)
		other	152.6 (1.6)
	Income in €	under 2000	3663.2 (38.3)
		2000–4000	3690.5 (38.6)
		over 4000	1339 (14)
		No Answer	880.1 (9.2)
	Relationship	Living Alone	2833.6 (29.6)
		Living with Partner	6680.3 (69.8)
		No Answer	59.1 (0.6)
	Migration	No Migration Background	8868.8 (92.6)
		Migration Background	704.2 (7.4)
Clinical	BMI	under 20	455.2 (4.8)
		20–25	3598.3 (37.6)
		over 25	5519.6 (57.7)
	Comorbidities	0	6099.1 (63.7)
		1–2	3069.3 (32.1)
		3+	404.7 (4.2)
	Depressive Symptoms	No	8654.4 (90.4)
		Yes	918.6 (9.6)
Urbanisation	DEGURBA	Thinly	521 (5.4)
		Intermediate	4226 (44.1)
		Densely	4826 (50.4)
	Noise	>55 dB	5458.6 (57.0)
**Category**	**Variable**	**Level**	**Mean (SD)**
Urbanisation	Green Area	Green area within 300 m radius (%)	38.7 (24.1)
	Green Distance	Distance to nearest green space (m)	51.5 (50.4)
	Population	People within same 100 m × 100 m square	74.4 (74.9)

ISCED = International Standard Classification of Education, BMI = Body Mass Index, DEGURBA = Degree of Urbanization, SD = Standard deviation.

**Table 2:** Shows the results for the univariate models 1a, 1b, 1c, and 1d from the main analysis (multiple imputation with Firth correction) without adjustment for DEGURBA and the confounders. Odds ratio, confidence intervals, and *p*-values are given adjusted and unadjusted for multiplicity.

		Unadjusted		Adjusted	
Variable	Level	OR (95% CI)	*p*-Value	OR (95% CI)	*p*-Value
Green Area	per 10,000 m^2^	0.98 (0.97, 0.99)	<0.001	0.98 (0.96, 1.00)	0.003
Green Distance	per 100 m	1.09 (0.94, 1.27)	0.241	1.09 (0.88, 1.37)	1
Population	per 100 People	1.36 (1.25, 1.49)	<0.001	1.36 (1.20, 1.55)	<0.001
Noise	No	1		1	
	Yes	0.94 (0.81, 1.09)	0.429	0.94 (0.76, 1.17)	1

OR = Odds ratio, CI = Confidence Interval.

**Table 3:** Shows the results for model 2 from the main analysis (multiple imputation with Firth correction), adjusted for DEGURBA and all variables. Odds ratio, confidence intervals, and *p*-values are given adjusted and unadjusted for multiplicity.

		Unadjusted		Adjusted	
Variable	Level	OR (95% CI)	*p*-Value	OR (95% CI)	*p*-Value
Green Area	per 10,000 m^2^	0.99 (0.97, 1.00)	0.098	0.99 (0.97, 1.01)	0.538
Green Distance	per 100 m	0.89 (0.74, 1.08)	0.232	0.89 (0.68, 1.17)	0.963
Population	per 100 People	1.34 (1.22, 1.47)	<0.001	1.34 (1.16, 1.53)	<0.001
Noise	No	1		1	
	Yes	0.97 (0.83, 1.12)	0.654	0.97 (0.77, 1.21)	1

OR = Odds ratio, CI = Confidence Interval.

**Table 4:** Shows the results for model 3 from the main analysis (multiple imputation with Firth correction) including adjustment for DEGURBA, all variables, and confounders. Odds ratio, confidence intervals, and *p*-values are given adjusted and unadjusted for multiplicity. *p*-values are not adjusted for multiplicity.

Domain	Variable	Level		OR (95% CI)	*p*-Value
Sociodemographic	Age	40–50		1	
		50–60		1.10 (0.91, 1.33)	0.323
		60–70		0.69 (0.55, 0.86)	0.001
	Sex	Female		1	
		Male		0.73 (0.62, 0.86)	<0.001
	Education	ISCED 0–2		1	
		ISCED 3–5		0.89 (0.67, 1.17)	0.403
		ISCED 6–8		0.81 (0.59, 1.12)	0.206
		other		1.05 (0.57, 1.95)	0.873
	Income in €	under 2000		1	
		2000–4000		0.81 (0.68, 0.96)	0.017
		over 4000		0.60 (0.45, 0.81)	0.001
		No Answer		0.97 (0.75, 1.27)	0.846
	Relationship	Living Alone		1	
		Living with Partner		0.68 (0.58, 0.80)	<0.001
		No Answer		0.46 (0.15, 1.37)	0.163
	Migration	No Migration Background		1	
		Migration Background		2.28 (1.74, 2.99)	<0.001
Clinical	BMI	under 20		1.22 (0.86, 1.73)	0.257
		20–25		1	
		over 25		1.18 (0.99, 1.40)	0.066
	Comorbidities	0		1	
		1–2		1.88 (1.59, 2.23)	<0.001
		3+		3.64 (2.68, 4.95)	<0.001
		**Unadjusted**		**Adjusted**	
**Variable**	**Level**	**OR (95% CI)**	***p*** **-Value**	**OR (95% CI)**	***p*** **-Value**
Green Area	per 10,000 m^2^	1.00 (0.98, 1.01)	0.549	1.00 (0.97, 1.02)	1
Green Distance	per 100 m	0.94 (0.78, 1.14)	0.549	0.94 (0.71, 1.25)	1
Population	per 100 People	1.15 (1.04, 1.27)	0.006	1.15 (0.99, 1.34)	0.070
Noise	≤55 dB	1		1	
	>55 dB	0.89 (0.76, 1.03)	0.0126	0.89 (0.71, 1.12)	0.652

OR = Odds ratio, CI = Confidence Interval, ISCED = International Standard Classification of Education, BMI = Body Mass Index.

**Table 5:** Shows the variables green area, green distance, population density, and noise level within the three groups of study participants living in thinly, intermediate, or densely urbanized areas.

	Thinly	Intermediate	Densely
Green Area in %, mean (SD)	53.6 (20.8)	48.6 (24.4)	29.2 (19.5)
Green Distance in m, mean (SD)	28.5 (31.0)	39.8 (39.4)	63.1 (56.9)
Population N, mean (SD)	39.1 (31.4)	47.8 (46.0)	95.1 (82.8)
Noise > 55 dB, N (%)	292 (60.8)	2116 (54.7)	2313 (57.2)

SD = Standard deviation.

## Data Availability

Data can be requested at the corresponding author: v.frey@salk.at.

## Appendix A. Sensitivity Analyses

(1) Results for Models 1a to 1d for Complete Case Analysis.

**Table A1:** Shows the results for the univariate Models 1a, 1b, 1c, and 1d from the complete case analysis. Confidence intervals and *p*-values are given adjusted and unadjusted for multiplicity.

		Unadjusted		Adjusted	
Variable	Level	OR (95% CI)	*p*-Value	OR (95% CI)	*p*-Value
Green Area	per 10,000 m^2^	0.98 (0.97, 0.99)	0.003	0.98 (0.97, 1.00)	0.036
Green Distance	per 100 m	1.05 (0.91, 1.22)	0.505	1.05 (0.85, 1.30)	1
Population	per 100 People	1.30 (1.19, 1.42)	<0.001	1.30 (1.14, 1.48)	<0.001
Noise	≤55 dB	1		1	
	>55 dB	0.94 (0.81, 1.09)	0.429	0.94 (0.76, 1.17)	1

(2) Results for Model 2 for Complete Case Analysis.

**Table A2:** Shows the results for Model 2 from the complete case analysis. Confidence intervals and *p*-values are given adjusted and unadjusted for multiplicity.

		Unadjusted		Adjusted	
Variable	Level	OR (95% CI)	*p*-Value	OR (95% CI)	*p*-Value
Green Area	per 10,000 m^2^	0.99 (0.97, 1.00)	0.095	0.99 (0.97, 1.01)	0.561
Green Distance	per 100 m	0.88 (0.73, 1.05)	0.150	0.88 (0.66, 1.16)	0.765
Population	per 100 People	1.27 (1.16, 1.41)	<0.001	1.27 (1.10, 1.48)	<0.001
Noise	≤55 dB	1		1	
	>55 dB	0.92 (0.79, 1.08)	0.312	0.92 (0.73, 1.17)	1

(3) Results for Model 3 for Complete Case Analysis.

**Table A3:** Shows the results for Model 3 from the complete case analysis. Confidence intervals and *p*-values are given adjusted (for urbanization parameters) and unadjusted for multiplicity.

			Unadjusted		Adjusted	
Domain	Variable	Level	OR (95% CI)	*p*-Value	OR (95% CI)	*p*-Value
Socio- demographic	Age	40–50	1			
		50–60	1.07 (0.86, 1.29)	0.468		
		60–70	0.67 (0.53, 0.83)	<0.001		
	Sex	Female	1			
		Male	0.72 (0.61, 0.85)	<0.001		
	Education	ISCED 0–2	1			
		ISCED 3–5	0.90 (0.68, 1.19)	0.473		
		ISCED 6–8	0.83 (0.61, 1.14)	0.253		
		other	1.07 (0.57, 2.01)	0.826		
	Income in €	under 2000	1			
		2000–4000	0.81 (0.67, 0.97)	0.023		
		over 4000	0.60 (0.44, 0.81)	0.001		
		No Answer	0.94 (0.72, 1.23)	0.658		
	Relationship	Living Alone	1			
		Living with Partner	0.68 (0.58, 0.80)	<0.001		
		No Answer	0.40 (0.12, 1.31)	0.129		
	Migration	No Migration Background	1			
		Migration Background	2.08 (1.57, 2.76)	<0.001		
Clinical	BMI	under 20	1.23 (0.86, 1.75)	0.253		
		20–25	1			
		over 25	1.19 (1.00, 1.43)	0.051		
	Comorbidities	0	1			
		1–2	1.91 (1.61, 2.27)	<0.001		
		3+	3.57 (2.62, 4.88)	<0.001		
Urbanisation	Green Area	per 10,000 m^2^	0.99 (0.98, 1.01)	0.397	0.99 (0.97, 1.02)	1
	Green Distance	per 100 m	0.93 (0.77, 1.11)	0.424	0.93 (0.70, 1.23)	1
	Population	per 100 People	1.13 (1.02, 1.26)	0.021	1.13 (0.96, 1.33)	0.203
	Noise	≤55 dB	1		1	
		>55 dB	0.86 (0.74, 1.01)	0.062	0.86 (0.68, 1.10)	0.418

(4) Results for Models 1a to 1d for Complete Case Analysis with Firth Correction.

**Table A4:** Shows the results for the univariate Models 1a, 1b, 1c, and 1d from the complete case analysis with firth correction. Confidence intervals and *p*-values are given adjusted and unadjusted for multiplicity.

		Unadjusted		Adjusted	
Variable	Level	OR (95% CI)	*p*-Value	OR (95% CI)	*p*-Value
Green Area	per 10,000 m^2^	0.98 (0.97, 0.99)	0.003	0.98 (0.97, 1.00)	0.037
Green Distance	per 100 m	1.05 (0.91, 1.22)	0.486	1.05 (0.85, 1.31)	1
Population	per 100 People	1.30 (1.19, 1.42)	<0.001	1.30 (1.14, 1.48)	<0.001
Noise	≤55 dB	1		1	
	>55 dB	0.94, (0.81, 1.09)	0.427	0.94 (0.76, 1.17)	1

(5) Results for Model 2 for Complete Case Analysis with Firth Correction.

**Table A5:** Shows the results for Model 2 from the complete case analysis with firth correction. Confidence intervals and *p*-values are given adjusted and unadjusted for multiplicity.

		Unadjusted		Adjusted	
Variable	Level	OR (95% CI)	*p*-Value	OR (95% CI)	*p*-Value
Green Area	per 10,000 m^2^	0.99 (0.97, 1.00)	0.097	0.99 (0.97, 1.01)	0.569
Green Distance	per 100 m	0.88 (0.73, 1.05)	0.156	0.88 (0.66, 1.16)	0.787
Population	per 100 People	1.28 (1.16, 1.41)	<0.001	1.28 (1.10, 1.48)	<0.001
Noise	≤55 dB	1		1	
	>55 dB	0.92 (0.79, 1.08)	0.311	0.92 (0.73, 1.17)	1

(6) Results for Model 3 for Complete Case Analysis with Firth Correction.

**Table A6:** Shows the results for Model 3 from the complete case analysis with Firth correction. Confidence intervals and *p*-values are given adjusted (for urbanization parameters) and unadjusted for multiplicity.

			Unadjusted		Adjusted	
Domain	Variable	Level	OR (95% CI)	*p*-Value	OR (95% CI)	*p*-Value
Socio- demographic	Age	40–50	1			
		50–60	1.07 (0.89, 1.29)	0.472		
		60–70	0.67 (0.54, 0.83)	<0.001		
	Sex	Female	1			
		Male	0.72 (0.61, 0.85)	<0.001		
	Education	ISCED 0–2	1			
		ISCED 3–5	0.90 (0.68, 1.19)	0.451		
		ISCED 6–8	0.83 (0.61, 1.13)	0.242		
		other	1.10 (0.59, 2.03)	0.766		
	Income in €	under 2000	1			
		2000–4000	0.81 (0.67, 0.97)	0.023		
		over 4000	0.60 (0.45, 0.81)	0.001		
		No Answer	0.94 (0.72, 1.24)	0.680		
	Relationship	Living Alone	1			
		Living with Partner	0.68 (0.58, 0.80)	<0.001		
		No Answer	0.46 (0.15, 1.38)	0.165		
	Migration	No Migration Background	1			
		Migration Background	2.08 (1.58, 2.76)	<0.001		
Clinical	BMI	under 20	1.24 (0.87, 1.76)	0.231		
		20–25	1			
		over 25	1.19 (1.00, 1.42)	0.051		
	Comorbidities	0	1			
		1–2	1.91 (1.61, 2.26)	<0.001		
		3+	3.57 (2.62, 4.87)	<0.001		
Urbanisation	Green Area	per 10,000 m^2^	0.99 (0.98, 1.01)	0.403	0.99 (0.97, 1.02)	1
	Green Distance	per 100 m	0.93 (0.78, 1.12)	0.437	0.93 (0.70, 1.23)	1
	Population	per 100 People	1.13 (1.02, 1.26)	0.019	1.13 (0.96, 1.33)	0.192
	Noise	≤55 dB	1		1	
		>55 dB	0.86 (0.74, 1.01)	0.061	0.86 (0.68, 1.10)	0.414

(7) Results for Models 1a to 1d for Multiple Imputation Analysis without Firth Correction.

**Table A7:** Shows the results for the univariate Models 1a, 1b, 1c, and 1d from the multiple imputation analysis. Confidence intervals and *p*-values are given adjusted and unadjusted for multiplicity.

		Unadjusted		Adjusted	
Variable	Level	OR (95% CI)	*p*-Value	OR (95% CI)	*p*-Value
Green Area	per 10,000 m^2^	0.98 (0.97, 0.99)	<0.001	0.98 (0.96, 1.00)	0.003
Green Distance	per 100 m	1.09 (0.94, 1.27)	0.250	1.09 (0.88, 1.36)	1
Population	per 100 People	1.36 (1.25, 1.49)	<0.001	1.36 (1.20, 1.55)	<0.001
Noise	≤55 dB	1		1	
	>55 dB	0.99 (0.86, 1.14)	0.877	0.99 (0.80, 1.22)	1

(8) Results for Model 2 for Multiple Imputation Analysis without Firth Correction.

**Table A8:** Shows the results for Model 2 from the multiple imputation analysis. Confidence intervals and *p*-values are given adjusted and unadjusted for multiplicity.

		Unadjusted		Adjusted	
Variable	Level	OR (95% CI)	*p*-Value	OR (95% CI)	*p*-Value
Green Area	per 10,000 m^2^	0.99 (0.97, 1.00)	0.096	0.99 (0.97, 1.01)	0.531
Green Distance	per 100 m	0.89 (0.74, 1.07)	0.225	0.89 (0.68, 1.17)	0.942
Population	per 100 People	1.34 (1.22, 1.47)	<0.001	1.34 (1.16, 1.53)	<0.001
Noise	≤55 dB	1		1	
	>55 dB	0.97 (0.83, 1.12)	0.656	0.97 (0.77, 1.21)	1

(9) Results for Model 3 for Multiple Imputation Analysis without Firth Correction.

**Table A9:** Shows the results for Model 3 from the multiple imputation analysis. Confidence intervals and *p*-values are given adjusted (for urbanization parameters) and unadjusted for multiplicity.

			Unadjusted		Adjusted	
Domain	Variable	Level	OR (95% CI)	*p*-Value	OR (95% CI)	*p*-Value
Socio- demographic	Age	40–50	1			
		50–60	1.10 (0.91, 1.33)	0.320		
		60–70	0.69 (0.55, 0.86)	0.001		
	Sex	Female	1			
		Male	0.73 (0.62, 0.86)	<0.001		
	Education	ISCED 0–2	1			
		ISCED 3–5	0.89 (0.67, 1.18)	0.416		
		ISCED 6–8	0.81 (0.59, 1.12)	0.211		
		other	1.03 (0.55, 1.94)	0.920		
	Income in €	under 2000	1			
		2000–4000	0.81 (0.68, 0.96)	0.017		
		over 4000	0.60 (0.44, 0.81)	0.001		
		No Answer	0.97 (0.74, 1.27)	0.827		
	Relationship	Living Alone	1			
		Living with Partner	0.68 (0.58, 0.80)	<0.001		
		No Answer	0.40 (0.12, 1.33)	0.135		
	Migration	No Migration Background	1			
		Migration Background	2.29 (1.74, 3.00)	<0.001		
Clinical	BMI	under 20	1.21 (0.86, 1.72)	0.278		
		20–25	1			
		over 25	1.18 (0.99, 1.40)	0.066		
	Comorbidities	0	1			
		1–2	1.89 (1.59, 2.23)	<0.001		
		3+	3.65 (2.68, 4.96)	<0.001		
Urbanisation	Green Area	per 10,000 m^2^	1.00 (0.98, 1.01)	0.544	1.00 (0.97, 1.02)	1
	Green Distance	per 100 m	0.94 (0.78, 1.14)	0.538	0.94 (0.71, 1.25)	1
	Population	per 100 People	1.15 (1.04, 1.27)	0.006	1.15 (0.99, 1.34)	0.074
	Noise	≤55 dB	1		1	
		>55 dB	0.89 (0.76, 1.03)	0.128	0.89 (0.71, 1.12)	0.656
